# 2-Vinyl­pyridine–tris­(penta­fluoro­phen­yl)borane hexane monosolvate

**DOI:** 10.1107/S1600536812013153

**Published:** 2012-03-31

**Authors:** Marcus Klahn, Anke Spannenberg, Uwe Rosenthal

**Affiliations:** a Leibniz-Institut für Katalyse e. V. an der Universität Rostock, Albert-Einstein-Strasse 29a, 18059 Rostock, Germany

## Abstract

The title compound, C_7_H_7_N·B(C_6_F_5_)_3_·C_6_H_14_, was obtained by the stoichiometric reaction of 2-vinyl­pyridine and tris­(penta­fluoro­phen­yl)borane in toluene. The formed adduct exhibits a restricted rotation along the B—N bond resulting in an asymmetry, which can be also observed in the ^19^F NMR spectra. The B—N distance is equivalent to the distances found for 2-methyl­pyridine and 2-ethyl­pyridine B(C_6_F_5_)_3_ adducts. For the final refinement, the contributions of disordered solvent mol­ecules were removed from the diffraction data with SQUEEZE in *PLATON* [van der Sluis & Spek (1990). *Acta Cryst.* A**46**, 194–201; Spek (2009). *Acta Cryst.* D**65**, 148–155].

## Related literature

For general aspects of related compounds, see: Focante *et al.* (2006 ▶); Stephan & Erker (2010 ▶); Welch *et al.* (2007 ▶). For related structures, see: Geier *et al.* (2009 ▶). For the use of SQUEEZE in *PLATON* to remove the contributions of disordered solvent mol­ecules, see: van der Sluis & Spek (1990 ▶); Spek (2009 ▶).
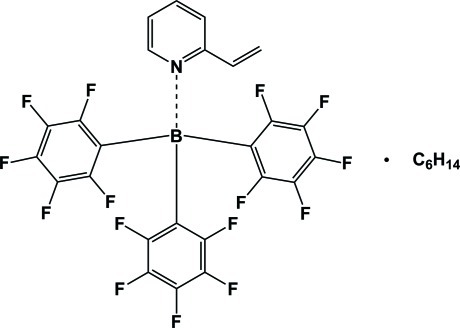


## Experimental

### 

#### Crystal data

C_25_H_7_BF_15_N·C_6_H_14_
*M* *_r_* = 730.30
Monoclinic, 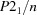

*a* = 12.4173 (2) Å
*b* = 17.1269 (3) Å
*c* = 13.9211 (2) Å
β = 100.889 (1)°
*V* = 2907.29 (8) Å^3^
*Z* = 4
Mo *K*α radiation
μ = 0.16 mm^−1^
*T* = 150 K
0.35 × 0.33 × 0.25 mm

#### Data collection

Bruker Kappa APEXII DUO diffractometer
Absorption correction: multi-scan (*SADABS*; Bruker, 2008 ▶) *T*_min_ = 0.695, *T*_max_ = 0.746
55747 measured reflections
6929 independent reflections
5324 reflections with *I* > 2σ(*I*)
*R*_int_ = 0.030

#### Refinement

*R*[*F* ^2^ > 2σ(*F* ^2^)] = 0.047
*wR*(*F* ^2^) = 0.113
*S* = 1.04
6929 reflections
379 parameters
H-atom parameters constrained
Δρ_max_ = 0.22 e Å^−3^
Δρ_min_ = −0.25 e Å^−3^

### 

Data collection: *APEX2* (Bruker, 2011 ▶); cell refinement: *SAINT* (Bruker, 2009 ▶); data reduction: *SAINT*; program(s) used to solve structure: *SHELXS97* (Sheldrick, 2008 ▶); program(s) used to refine structure: *SHELXL97* (Sheldrick, 2008 ▶); molecular graphics: *XP* in *SHELXTL* (Sheldrick, 2008 ▶); software used to prepare material for publication: *SHELXTL*.

## Supplementary Material

Crystal structure: contains datablock(s) I, global. DOI: 10.1107/S1600536812013153/vm2164sup1.cif

Structure factors: contains datablock(s) I. DOI: 10.1107/S1600536812013153/vm2164Isup2.hkl

Additional supplementary materials:  crystallographic information; 3D view; checkCIF report

## Figures and Tables

**Fig. 1.**
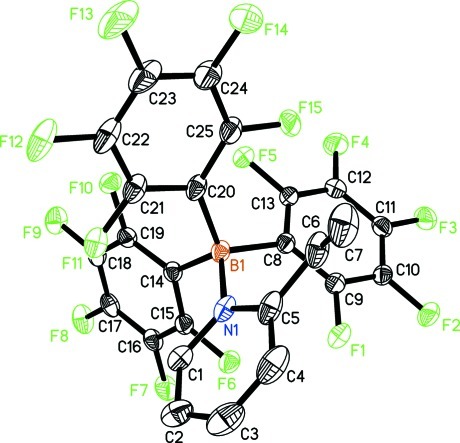
The molecular structure of the title compound with the atom labels and 30% displacement ellipsoids. Hydrogen atoms are omitted for clarity.

## supplementary crystallographic information

### Comment

The concept of "Frustrated Lewis pairs" has been put forth and coined by Welch *et al.* (2007). The key feature is the sterical hindrance between the Lewis acids and bases preventing the formation of a classical adduct which gives rise to a unique reactivity due to the interaction of the basic and acidic centers. This characteristic facilitates a lot of reactions with a wide variety of reagents, as it has been overviewed by Stephan & Erker (2010). The substrates can be either small molecules like H_2_, CO_2_, and N_2_O, or larger ones like terminal olefins, alkynes, dienes, B–H bonds, disulfides and the C–O bonds of cyclic ethers. The conversion of 2-vinylpyridine with tris(pentafluorophenyl)borane proceeds similar to the general formation of Lewis acid base adducts presented before by Focante *et al.* (2006) and Geier *et al.* (2009). The nature of the adduct (figure 1) is confirmed by the precluded activation of dihydrogen. As found for other pyridine derivatives, the compound gives ^19^F-NMR spectra with 15 signals, one for each fluorine atom at the borane. This asymmetry results from a restricted rotation of the B–N bond. The B–N distance of the title compound (1.637 (3) Å) is found to be the same as for the analogous compounds 2-methylvinylpyridine/B(C_6_F_5_)_3_ 1.639 (2) Å and 2-ethylpyridine/B(C_6_F_5_)_3_ 1.638 (2) Å, respectively (Geier *et al.*, 2009). This finding corroborates the assumption that the vinyl group in *ortho*-position to the nitrogen has no influence on the bonding situation. The boron center exhibits a distorted tetrahedral coordination geometry.

### Experimental

Solid tris(pentafluorophenyl)borane (0.512 g, 1.0 mmol) and vinylpyridine (0.11 ml, 1.0 mmol) were dissolved in 25 ml of toluene resulting in a colorless solution. The reaction was allowed to stir for 2 h at 40 °C. The solvent was removed *in vaccuo* and the residue was extracted with *n*-hexane. Slow evaporation of the solvent resulted in the formation of colorless prisms. Isolated yield for pure crystalline material 0.380 g (62%).

### Refinement

For the final refinement the contributions of disordered solvent molecules were removed from the diffraction data with *PLATON* / SQUEEZE (van der Sluis & Spek, 1990; Spek, 2009). SQUEEZE estimated the electron counts in the voids of 196 Å^3^ to be 53. H atoms were placed in idealized positions with d(C—H) = 0.95 Å and refined using a riding model with *U*_iso_(H) fixed at 1.2 *U*_eq_(C).

### Crystal data

**Table:**

C_25_H_7_BF_15_N·C_6_H_14_	*F*(000) = 1416
*M**_r_* = 703.30	*D*_x_ = 1.607 Mg m^−^^3^
Monoclinic, *P*2_1_/*n*	Mo *K*α radiation, λ = 0.71073 Å
*a* = 12.4173 (2) Å	Cell parameters from 9876 reflections
*b* = 17.1269 (3) Å	θ = 2.3–26.3°
*c* = 13.9211 (2) Å	µ = 0.16 mm^−^^1^
β = 100.889 (1)°	*T* = 150 K
*V* = 2907.29 (8) Å^3^	Prism, colourless
*Z* = 4	0.35 × 0.33 × 0.25 mm

### Data collection

**Table:**

Bruker Kappa APEXII DUO diffractometer	6929 independent reflections
Radiation source: fine-focus sealed tube	5324 reflections with *I* > 2σ(*I*)
Curved graphite monochromator	*R*_int_ = 0.030
Detector resolution: 8.3333 pixels mm^-1^	θ_max_ = 27.9°, θ_min_ = 1.9°
ω scans	*h* = −16→16
Absorption correction: multi-scan (*SADABS*; Bruker, 2008)	*k* = 0→22
*T*_min_ = 0.695, *T*_max_ = 0.746	*l* = 0→18
55747 measured reflections	

### Refinement

**Table:**

Refinement on *F*^2^	Primary atom site location: structure-invariant direct methods
Least-squares matrix: full	Secondary atom site location: difference Fourier map
*R*[*F*^2^ > 2σ(*F*^2^)] = 0.047	Hydrogen site location: inferred from neighbouring sites
*wR*(*F*^2^) = 0.113	H-atom parameters constrained
*S* = 1.04	*w* = 1/[σ^2^(*F*_o_^2^) + (0.0312*P*)^2^ + 2.2216*P*] where *P* = (*F*_o_^2^ + 2*F*_c_^2^)/3
6929 reflections	(Δ/σ)_max_ < 0.001
379 parameters	Δρ_max_ = 0.22 e Å^−^^3^
0 restraints	Δρ_min_ = −0.25 e Å^−^^3^

### Special details

**Table:**

Geometry. All e.s.d.'s (except the e.s.d. in the dihedral angle between two l.s. planes) are estimated using the full covariance matrix. The cell e.s.d.'s are taken into account individually in the estimation of e.s.d.'s in distances, angles and torsion angles; correlations between e.s.d.'s in cell parameters are only used when they are defined by crystal symmetry. An approximate (isotropic) treatment of cell e.s.d.'s is used for estimating e.s.d.'s involving l.s. planes.
Refinement. Refinement of *F*^2^ against ALL reflections. The weighted *R*-factor *wR* and goodness of fit *S* are based on *F*^2^, conventional *R*-factors *R* are based on *F*, with *F* set to zero for negative *F*^2^. The threshold expression of *F*^2^ > σ(*F*^2^) is used only for calculating *R*-factors(gt) *etc*. and is not relevant to the choice of reflections for refinement. *R*-factors based on *F*^2^ are statistically about twice as large as those based on *F*, and *R*- factors based on ALL data will be even larger.

### Fractional atomic coordinates and isotropic or equivalent isotropic displacement parameters (Å^2^)

**Table:**

	*x*	*y*	*z*	*U*_iso_*/*U*_eq_	
C1	0.90231 (17)	0.13181 (12)	0.38660 (15)	0.0434 (5)	
H1	0.8263	0.1383	0.3864	0.052*	
C2	0.9757 (2)	0.16041 (15)	0.46482 (18)	0.0578 (6)	
H2	0.9512	0.1865	0.5169	0.069*	
C3	1.0860 (2)	0.15025 (18)	0.4657 (2)	0.0713 (8)	
H3	1.1392	0.1673	0.5200	0.086*	
C4	1.11785 (19)	0.11521 (16)	0.3873 (2)	0.0668 (8)	
H4	1.1938	0.1084	0.3874	0.080*	
C5	1.04135 (16)	0.08929 (12)	0.30710 (19)	0.0493 (5)	
C6	1.07643 (17)	0.05611 (14)	0.2212 (2)	0.0565 (6)	
H6	1.0328	0.0165	0.1853	0.068*	
C7	1.1666 (2)	0.07912 (17)	0.1916 (3)	0.0765 (9)	
H7A	1.2114	0.1187	0.2265	0.092*	
H7B	1.1865	0.0561	0.1353	0.092*	
C8	0.84554 (14)	−0.02844 (11)	0.19742 (13)	0.0334 (4)	
C9	0.90655 (14)	−0.08385 (11)	0.25654 (13)	0.0343 (4)	
C10	0.90397 (16)	−0.16275 (11)	0.23593 (14)	0.0375 (4)	
C11	0.83804 (18)	−0.18957 (11)	0.15249 (15)	0.0415 (4)	
C12	0.77312 (18)	−0.13771 (13)	0.09240 (15)	0.0449 (5)	
C13	0.77700 (16)	−0.05962 (12)	0.11631 (14)	0.0391 (4)	
C14	0.71442 (14)	0.07074 (10)	0.25130 (13)	0.0313 (4)	
C15	0.69487 (15)	0.02992 (11)	0.33276 (13)	0.0346 (4)	
C16	0.59468 (16)	0.02570 (11)	0.36130 (15)	0.0389 (4)	
C17	0.50623 (15)	0.06250 (11)	0.30572 (15)	0.0396 (4)	
C18	0.51920 (15)	0.10188 (11)	0.22336 (15)	0.0385 (4)	
C19	0.62162 (15)	0.10543 (10)	0.19769 (13)	0.0334 (4)	
C20	0.85181 (15)	0.12444 (12)	0.13074 (16)	0.0412 (5)	
C21	0.83817 (15)	0.20401 (12)	0.14261 (17)	0.0454 (5)	
C22	0.85464 (18)	0.25980 (14)	0.0756 (2)	0.0596 (7)	
C23	0.8865 (2)	0.23690 (17)	−0.0090 (2)	0.0714 (8)	
C24	0.9022 (2)	0.15996 (17)	−0.0244 (2)	0.0709 (8)	
C25	0.88500 (18)	0.10540 (14)	0.04498 (18)	0.0539 (6)	
N1	0.93177 (12)	0.09495 (9)	0.30993 (13)	0.0385 (4)	
B1	0.83605 (16)	0.06510 (12)	0.22003 (16)	0.0332 (4)	
F1	0.97508 (9)	−0.06271 (7)	0.33938 (8)	0.0438 (3)	
F2	0.96667 (11)	−0.21289 (7)	0.29565 (9)	0.0499 (3)	
F3	0.83723 (12)	−0.26568 (7)	0.12948 (9)	0.0543 (3)	
F4	0.70812 (13)	−0.16365 (9)	0.01098 (9)	0.0679 (4)	
F5	0.70978 (11)	−0.01194 (7)	0.05566 (8)	0.0511 (3)	
F6	0.77858 (9)	−0.00810 (7)	0.38990 (8)	0.0424 (3)	
F7	0.58340 (11)	−0.01383 (7)	0.44164 (9)	0.0528 (3)	
F8	0.40755 (10)	0.05864 (7)	0.33126 (10)	0.0553 (3)	
F9	0.43240 (9)	0.13657 (7)	0.16772 (10)	0.0515 (3)	
F10	0.62556 (9)	0.14561 (7)	0.11527 (8)	0.0405 (3)	
F11	0.80562 (10)	0.23048 (7)	0.22390 (10)	0.0492 (3)	
F12	0.83828 (12)	0.33561 (8)	0.09278 (14)	0.0760 (5)	
F13	0.90227 (15)	0.29033 (11)	−0.07537 (15)	0.1035 (7)	
F14	0.93402 (17)	0.13562 (12)	−0.10669 (14)	0.1034 (7)	
F15	0.90590 (12)	0.03096 (8)	0.02312 (10)	0.0670 (4)	

### Atomic displacement parameters (Å^2^)

**Table:**

	*U*^11^	*U*^22^	*U*^33^	*U*^12^	*U*^13^	*U*^23^
C1	0.0383 (10)	0.0435 (11)	0.0467 (11)	0.0014 (8)	0.0031 (9)	0.0103 (9)
C2	0.0560 (14)	0.0589 (14)	0.0517 (13)	−0.0058 (11)	−0.0068 (11)	0.0084 (11)
C3	0.0509 (14)	0.0776 (19)	0.0741 (18)	−0.0134 (13)	−0.0170 (13)	0.0157 (15)
C4	0.0320 (11)	0.0657 (16)	0.096 (2)	−0.0033 (11)	−0.0044 (12)	0.0250 (15)
C5	0.0305 (9)	0.0393 (11)	0.0777 (16)	0.0015 (8)	0.0091 (10)	0.0185 (10)
C6	0.0337 (10)	0.0484 (12)	0.0917 (18)	0.0077 (9)	0.0227 (11)	0.0178 (12)
C7	0.0389 (12)	0.0733 (18)	0.124 (3)	0.0028 (12)	0.0322 (15)	0.0183 (17)
C8	0.0294 (8)	0.0379 (9)	0.0360 (9)	0.0069 (7)	0.0145 (7)	0.0077 (8)
C9	0.0307 (9)	0.0378 (9)	0.0365 (10)	0.0022 (7)	0.0119 (7)	0.0071 (8)
C10	0.0409 (10)	0.0377 (10)	0.0375 (10)	0.0086 (8)	0.0165 (8)	0.0123 (8)
C11	0.0534 (12)	0.0357 (10)	0.0400 (10)	0.0042 (9)	0.0207 (9)	0.0020 (8)
C12	0.0511 (12)	0.0507 (12)	0.0336 (10)	0.0081 (9)	0.0096 (9)	−0.0024 (9)
C13	0.0413 (10)	0.0449 (11)	0.0332 (9)	0.0138 (8)	0.0123 (8)	0.0080 (8)
C14	0.0296 (8)	0.0315 (9)	0.0343 (9)	0.0015 (7)	0.0101 (7)	0.0002 (7)
C15	0.0338 (9)	0.0348 (9)	0.0368 (10)	0.0047 (7)	0.0111 (7)	0.0005 (7)
C16	0.0441 (10)	0.0348 (9)	0.0437 (11)	−0.0004 (8)	0.0230 (9)	−0.0002 (8)
C17	0.0302 (9)	0.0402 (10)	0.0530 (12)	−0.0028 (8)	0.0194 (8)	−0.0107 (9)
C18	0.0281 (9)	0.0361 (10)	0.0503 (11)	0.0018 (7)	0.0049 (8)	−0.0075 (8)
C19	0.0331 (9)	0.0334 (9)	0.0336 (9)	−0.0001 (7)	0.0060 (7)	−0.0043 (7)
C20	0.0300 (9)	0.0426 (10)	0.0546 (12)	0.0111 (8)	0.0174 (8)	0.0184 (9)
C21	0.0282 (9)	0.0458 (11)	0.0643 (14)	0.0062 (8)	0.0141 (9)	0.0174 (10)
C22	0.0381 (11)	0.0483 (13)	0.0928 (19)	0.0064 (9)	0.0130 (12)	0.0346 (13)
C23	0.0501 (14)	0.0793 (18)	0.092 (2)	0.0110 (13)	0.0333 (14)	0.0541 (16)
C24	0.0630 (15)	0.0844 (19)	0.0793 (18)	0.0288 (14)	0.0497 (14)	0.0443 (15)
C25	0.0466 (12)	0.0598 (14)	0.0642 (14)	0.0221 (10)	0.0332 (11)	0.0265 (11)
N1	0.0287 (7)	0.0349 (8)	0.0510 (10)	0.0023 (6)	0.0047 (7)	0.0121 (7)
B1	0.0282 (9)	0.0343 (10)	0.0386 (11)	0.0050 (8)	0.0101 (8)	0.0059 (8)
F1	0.0396 (6)	0.0386 (6)	0.0494 (7)	0.0036 (5)	−0.0013 (5)	0.0087 (5)
F2	0.0609 (8)	0.0361 (6)	0.0505 (7)	0.0103 (5)	0.0047 (6)	0.0115 (5)
F3	0.0768 (9)	0.0390 (6)	0.0494 (7)	0.0074 (6)	0.0176 (6)	−0.0017 (5)
F4	0.0918 (11)	0.0615 (9)	0.0424 (7)	0.0167 (8)	−0.0072 (7)	−0.0129 (6)
F5	0.0592 (7)	0.0535 (7)	0.0374 (6)	0.0205 (6)	0.0008 (5)	0.0042 (5)
F6	0.0417 (6)	0.0454 (6)	0.0428 (6)	0.0078 (5)	0.0148 (5)	0.0121 (5)
F7	0.0601 (8)	0.0505 (7)	0.0585 (8)	0.0043 (6)	0.0387 (6)	0.0083 (6)
F8	0.0366 (6)	0.0537 (7)	0.0840 (9)	−0.0013 (5)	0.0326 (6)	−0.0045 (7)
F9	0.0266 (5)	0.0561 (7)	0.0684 (8)	0.0036 (5)	0.0001 (5)	−0.0037 (6)
F10	0.0353 (6)	0.0488 (6)	0.0367 (6)	0.0071 (5)	0.0047 (4)	0.0059 (5)
F11	0.0452 (7)	0.0373 (6)	0.0649 (8)	0.0040 (5)	0.0100 (6)	0.0052 (6)
F12	0.0589 (9)	0.0427 (7)	0.1227 (14)	0.0026 (6)	0.0078 (9)	0.0343 (8)
F13	0.0922 (12)	0.1019 (13)	0.1287 (16)	0.0164 (10)	0.0526 (12)	0.0824 (12)
F14	0.1180 (15)	0.1220 (15)	0.0956 (13)	0.0472 (12)	0.0845 (12)	0.0574 (11)
F15	0.0828 (10)	0.0677 (9)	0.0652 (9)	0.0380 (8)	0.0518 (8)	0.0245 (7)

### Geometric parameters (Å, º)

**Table:**

C1—N1	1.349 (3)	C13—F5	1.345 (2)
C1—C2	1.372 (3)	C14—C19	1.383 (2)
C1—H1	0.9500	C14—C15	1.392 (2)
C2—C3	1.379 (4)	C14—B1	1.652 (3)
C2—H2	0.9500	C15—F6	1.351 (2)
C3—C4	1.368 (4)	C15—C16	1.377 (2)
C3—H3	0.9500	C16—F7	1.337 (2)
C4—C5	1.395 (4)	C16—C17	1.372 (3)
C4—H4	0.9500	C17—F8	1.340 (2)
C5—N1	1.372 (2)	C17—C18	1.366 (3)
C5—C6	1.462 (4)	C18—F9	1.341 (2)
C6—C7	1.325 (3)	C18—C19	1.386 (3)
C6—H6	0.9500	C19—F10	1.347 (2)
C7—H7A	0.9500	C20—C25	1.374 (3)
C7—H7B	0.9500	C20—C21	1.387 (3)
C8—C9	1.384 (2)	C20—B1	1.646 (3)
C8—C13	1.386 (3)	C21—F11	1.350 (3)
C8—B1	1.641 (3)	C21—C22	1.378 (3)
C9—F1	1.347 (2)	C22—F12	1.343 (3)
C9—C10	1.380 (3)	C22—C23	1.368 (4)
C10—F2	1.338 (2)	C23—F13	1.341 (3)
C10—C11	1.368 (3)	C23—C24	1.355 (4)
C11—F3	1.342 (2)	C24—F14	1.346 (3)
C11—C12	1.373 (3)	C24—C25	1.390 (3)
C12—F4	1.337 (2)	C25—F15	1.347 (3)
C12—C13	1.377 (3)	N1—B1	1.637 (3)
			
N1—C1—C2	123.8 (2)	F6—C15—C16	116.19 (16)
N1—C1—H1	118.1	F6—C15—C14	119.25 (15)
C2—C1—H1	118.1	C16—C15—C14	124.56 (17)
C1—C2—C3	118.2 (3)	F7—C16—C17	120.35 (17)
C1—C2—H2	120.9	F7—C16—C15	120.71 (18)
C3—C2—H2	120.9	C17—C16—C15	118.94 (17)
C4—C3—C2	119.0 (2)	F8—C17—C18	120.38 (18)
C4—C3—H3	120.5	F8—C17—C16	120.16 (18)
C2—C3—H3	120.5	C18—C17—C16	119.44 (16)
C3—C4—C5	121.5 (2)	F9—C18—C17	119.61 (17)
C3—C4—H4	119.2	F9—C18—C19	120.54 (18)
C5—C4—H4	119.2	C17—C18—C19	119.85 (17)
N1—C5—C4	118.9 (2)	F10—C19—C14	121.24 (16)
N1—C5—C6	120.1 (2)	F10—C19—C18	115.15 (16)
C4—C5—C6	121.0 (2)	C14—C19—C18	123.61 (17)
C7—C6—C5	122.6 (3)	C25—C20—C21	113.59 (18)
C7—C6—H6	118.7	C25—C20—B1	127.43 (18)
C5—C6—H6	118.7	C21—C20—B1	118.83 (18)
C6—C7—H7A	120.0	F11—C21—C22	116.2 (2)
C6—C7—H7B	120.0	F11—C21—C20	119.50 (17)
H7A—C7—H7B	120.0	C22—C21—C20	124.3 (2)
C9—C8—C13	113.76 (17)	F12—C22—C23	120.6 (2)
C9—C8—B1	127.32 (17)	F12—C22—C21	120.2 (3)
C13—C8—B1	118.49 (16)	C23—C22—C21	119.2 (2)
F1—C9—C10	115.29 (16)	F13—C23—C24	120.7 (3)
F1—C9—C8	120.73 (17)	F13—C23—C22	120.1 (3)
C10—C9—C8	123.98 (18)	C24—C23—C22	119.3 (2)
F2—C10—C11	119.78 (17)	F14—C24—C23	120.7 (2)
F2—C10—C9	120.75 (18)	F14—C24—C25	119.5 (3)
C11—C10—C9	119.46 (17)	C23—C24—C25	119.9 (2)
F3—C11—C10	120.35 (18)	F15—C25—C20	121.54 (18)
F3—C11—C12	120.40 (19)	F15—C25—C24	114.7 (2)
C10—C11—C12	119.25 (18)	C20—C25—C24	123.7 (2)
F4—C12—C11	119.51 (19)	C1—N1—C5	118.33 (18)
F4—C12—C13	121.04 (19)	C1—N1—B1	119.01 (15)
C11—C12—C13	119.44 (19)	C5—N1—B1	122.47 (18)
F5—C13—C12	116.65 (18)	N1—B1—C8	112.56 (14)
F5—C13—C8	119.34 (17)	N1—B1—C20	102.23 (15)
C12—C13—C8	124.01 (18)	C8—B1—C20	115.78 (16)
C19—C14—C15	113.56 (16)	N1—B1—C14	110.38 (15)
C19—C14—B1	126.37 (16)	C8—B1—C14	102.13 (14)
C15—C14—B1	119.61 (15)	C20—B1—C14	114.08 (14)
